# Field Calibrations of Soil Moisture Sensors in a Forested Watershed

**DOI:** 10.3390/s110606354

**Published:** 2011-06-16

**Authors:** Farhat Abbas, Ali Fares, Samira Fares

**Affiliations:** Natural Resources and Environmental Management Department, University of Hawaii-Manoa, Honolulu, HI 96822, USA; E-Mails: farhat@hawaii.edu (F.A.); sfares@hawaii.edu (S.F.)

**Keywords:** variable soil properties, soil water content, sensor field calibration, tropical soils

## Abstract

Spatially variable soil properties influence the performance of soil water content monitoring sensors. The objectives of this research were to: (i) study the spatial variability of bulk density (*ρ*_b_), total porosity (*θ*_t_), clay content (CC), electrical conductivity (EC), and pH in the upper Mākaha Valley watershed soils; (ii) explore the effect of variations in *ρ*_b_ and *θ*_t_ on soil water content dynamics, and (iii) establish field calibration equations for EC-20 (Decagon Devices, Inc), ML2x (Delta-T-Devices), and SM200 (Delta-T-Devices) sensors to mitigate the effect of soil spatial variability on their performance. The studied soil properties except pH varied significantly (*P* < 0.05) across the soil water content monitoring depths (20 and 80 cm) and six locations. There was a linear positive and a linear inverse correlation between the soil water content at sampling and *ρ*_b_, and between the soil water content at sampling and *θ*_t_, respectively. Values of laboratory measured actual *θ*_t_ correlated (*r* = 0.75) with those estimated from the relationship *θ*_t_ = 1 − *ρ*_b_/*ρ*_s_, where *ρ*_s_ is the particle density. Variations in the studied soil properties affected the performance of the default equations of the three tested sensors; they showed substantial under-estimations of the actual water content. The individual and the watershed-scale field calibrations were more accurate than their corresponding default calibrations. In conclusion, the sensors used in this study need site-specific calibrations in order to mitigate the effects of varying properties of the highly weathered tropical soils.

## 1. Introduction

Non-agricultural forested lands exhibit spatially variable soil water content as a function of soil basic properties [1], land cover [2], and topography [3]. Soil water content varies across a soil profile due to changes in *ρ*_b_, *θ*_t_, and CC [4,5]. Surface soil layers of forested watersheds are subject to higher soil water content dynamics due to evapotranspiration and rainfall; deep soil profiles have higher water content due to uniform conditions.

Accurate measurement of soil water content is important for water balance and hydrologic flux calculations, rainfall-runoff-infiltration models, ground truthing of remote sensing data, irrigation scheduling, water allocation calculations, and evaluation of potential drought impacts on stream flow [6]. Reliable measurement of soil water content with water sensors has been challenging in forested lands due to spatially variable soil physical and hydrological properties [6]. Variations in *ρ*_b_ have greater effects on sensor readings than those caused by CC or organic matter content [7].

Direct measurements of soil water content by the thermo-gravimetric method is more accurate than any other indirect method; however, this method is labor intensive, time consuming, destructive, and discrete for repetitive measurements. Indirect techniques of soil water content measurement (e.g., single- and multi-capacitance soil water content monitoring systems) overcome these disadvantages of the thermo-gravimetric method; in addition, they enable automate real-time spatially-distributed data collection [8,9]. Such techniques have been used for real-time monitoring of soil water content at different scales, *i.e*., greenhouse, field plots, watersheds subject to different agricultural management practices. Installation of theses sensors begins with a careful selection of monitoring locations, which are conceptually subdivided into macro- and micro-zones [8]. Macro-zone refers to the selection of one or several locations in a watershed or in an agricultural field characterized by dominant topography, soil type, vegetation, and management practices. On the other hand, micro-zone selection aims at determining the position of the sensor in relation to individual points/locations, soil depths (shallow *vs.* deep) or irrigation delivery points (drip or sprinkler emitter).

The single soil water content monitoring sensors EC-20 [10], ML2x [11], and SM200 [11] have been used in agricultural and non-agricultural settings. These sensors were calibrated under different soil and environmental conditions [12,13]. Czarnomski *et al.* [14] compared the accuracy and precision of the EC-20 with those of a TDR under field and laboratory conditions. They found that the default calibration equation of EC-20 underestimated water content by up to 0.12 cm^3^ cm^−3^ and that EC-20 wasn’t sensitive to *ρ*_b_; they also concluded that the EC-20 data were more consistent than those of TDR. Logsdon and Hornbuckle [15] reported that the larger measurement volume of the CS616 resulted in less spatial variability of soil water content compared to that of the ML2x because of the relatively smaller measurement volume of the latter. The use of default calibration equations results in considerable over- and under-estimations of soil water content measured by the EC-20 and ML2x, respectively [12]. These findings strongly recommend site-specific calibrations to improve the accuracy and performance of soil water content monitoring devices. Hu *et al.* [16] calibrated CS616, ML2x, and SM200 units and reported that the ML2x performed better than the other two sensors with new calibration equations.

**Figure 1.**
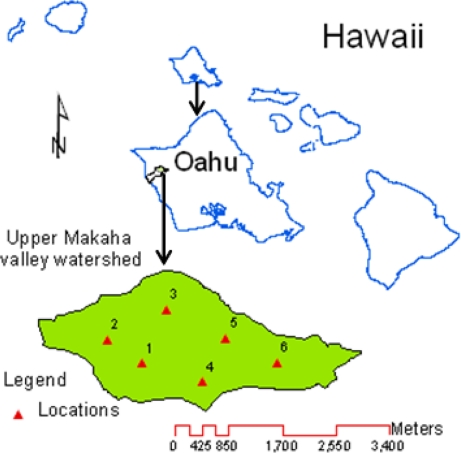
A map showing the upper Mākaha Valley sub-watershed and the six monitoring locations of the field calibration from which soil samples were collected and used to determine bulk density, total porosity, clay content, electrical conductivity and pH.

The Mākaha Valley watershed is located in the dry leeward side of the island of O’ahu, HI, USA (Figure 1). This watershed has been the home of a long-term hydrologic study aiming at determining the effects of rainfall variability, groundwater pumping, and invasive species on its hydrology and water quality [17]. The watershed has been instrumented with EC-20, ML2x, and SM200 sensors, and other equipment for real-time monitoring of water budget components. The objectives of this study were to (i) study the spatial variability of *ρ*_b_, *θ*_t_, CC, EC, and pH of the upper Mākaha Valley watershed soil, (ii) explore the effect of variations in *ρ*_b_ and *θ*_t_ on soil water content dynamics, and (iii) establish field calibration equations for EC-20, ML2x, and SM200 sensors to improve their performance.

## 2. Materials and Methods

### 2.1. Soil Water Content Sensing Devices

The sensors calibrated during this study were the EC-20, ML2x, and SM200 (Table 1). The calibration equation of the EC-20 is a linear function that relates EC-20 readings (V) to the actual soil water content. There is a linear correlation between soil water content measured with the ML2x and the square root of the dielectric constant (√*ɛ*) [18,19]. The SM200 probe has a similar calibration equation as that of the ML2x.

### 2.2. Experimental Locations

Field calibrations of the selected sensors were performed at two depths of six monitoring locations across the upper Mākaha Valley sub-watershed (Figure 1). These locations, referred to as locations 1 through 6 from here onward, were initially selected to represent the spatial variations of the topography, soil, and vegetation covers across the study area (Table 2). Soils in the lower valley are less permeable than those along the valley ridges. The soils over the valley floor and along the southeastern ridge of the upper valley are mainly clay loam, silty loam, and silty clay [20].

### 2.3. Soil Properties

Disturbed and undisturbed soil core samples were collected in three replicates from each depth of the six locations. The undisturbed soil core samples (radius = 2.5 cm; height = 7.5 cm) were collected with a sludge hammer soil sampler (Soilmoisture Equipment Crop. Santa Barbara, CA, USA). The trimmed soil cores were sealed with plastic caps, placed in labeled zip-lock plastic bags, and taken to the laboratory to measure their *ρ*_b_ and *θ*_t_ following the standard procedures described by Grossman and Reinsch [21] and Flint and Flint [22], respectively.

Replicates of the disturbed bulk soil samples were thoroughly mixed to produce a representative sample for each depth and location. These samples were air dried and sieved (<2 mm); a sub-sample was used to determine particle size distribution using the hydrometer method [23]. The textural triangle of the United States Department of Agriculture (USDA) classification scheme was used to determine the soil particle size distributions. Color schemes were used to determine the NRCS (Natural Resources Conservation Services) soil series information. EC and pH of these samples were measured from their 1:2 soil:water solutions with a multi-functional sympHony^®^ meter (Model SB90M5; Batavia, IL, USA) and the respective electrodes.

### 2.4. Calibration Procedure

Two units of each EC-20, ML2x, and SM200 sensor were installed at 20 and 80 cm depths at locations 1 through 6 following standard procedures [8]. Various soil water content levels ranging between field capacity (∼0.23 cm^3^ cm^−3^) and saturation (∼0.59 cm^3^ cm^−3^) were generated by intermittently applying water at and surrounding the sensor installation points. Watermark soil matric potential sensors were used to monitor the soil water potential at various soil water content levels. The soil water sensors and matric potential sensors were logged with their corresponding data loggers at 1-min intervals. At least 3 uniform sensor readings were averaged and used with the corresponding actual water content determined in the laboratory from intact soil core samples collected in three replicates from the close proximity of the sensors’ zone of influence such that the center of the soil cores aligned with the center of the individual sensor. These samples were used to determine actual soil water content following the thermo-gravimetric method. These samples were also used to determine *ρ*_b_ and *θ*_t_. Total porosity was also estimated from the following equation:

(1) $$\theta_{\text{t}}=1-\frac{\rho_{\text{b}}}{\rho_{\text{s}}}$$

where *ρ*_s_ is the particle density (2.65 g cm^−3^).

### 2.5. Calibration Equations and Data Analyses

Values of actual soil water content were plotted versus the respective readings (V) of the EC-20, ML2x, and SM200 to establish field calibration equations separately for the two depths (20 and 80 cm) at each location. A factorial analysis of variance (ANOVA) was conducted to evaluate the effect of soil depths and locations on *ρ*_b_, *θ*_t_, CC, EC, and pH using Statistix software package [24]. The coefficient of correlation (*r*), which represents the degree of association between the calculated and the actual water content; the root mean square error (RMSE), which represents the accuracy of calibration equation to predict actual water content; and the mean bias error (MBE), which is an indicator of sensor’s accuracy in form of the difference between means of the calculated and actual water contents, were used to evaluate the field and default calibration equations. The RMSE (cm^3^ cm^−3^) and MBE (cm^3^ cm^−3^) were calculated as follows:

(2) $$\text{RMSE}=\sqrt{\sum_{i=1}^{n}{\left(\theta_{ci}-\theta_{ai}\right)}^{2}/n}$$

(3) $$\text{MBE}=\sum_{i=1}^{n}(\theta_{ci}-\theta_{ai})/n$$

where *θ_ci_* and *θ_ai_* are the calculated and actual water content in cm^3^ cm^−3^, respectively, and *n* is the number of observations. Positive and negative values of MBE indicate over-estimation and under-estimation of the actual water content by the sensor, respectively. Larger *r* and smaller RMSE and MBE represent high sensor accuracy and vice versa.

## 3. Results and Discussion

### 3.1. Selected Soil Properties

Across the six soil water content monitoring locations, the soils at 20 cm depth had larger CC than those at 80 cm depth (Table 3). Clay content at 20 cm depth ranged from 157 at location 6 to 610 g kg^−1^ at location 2; whereas, at 80 cm depth, the CC values ranged from 288 at locations 4 and 5 to 690 g kg^−1^ at location 2. Soil from both depths at locations 1 and 4 were clay loam. Locations 2 and 3 had clay soils at both depths; whereas, at location 5, loam and sandy loam soils existed at 20 and 80 cm soil depths, respectively. Soil from the 80 cm depth at location 6 was silty clay. Soils at location 6 had the smallest CC among the other sampling locations. Different NRCS soil series were found across the watershed. Location 1 had Molisol, location 2 had Inceptisol, and locations 3 through 5 had Ultisol. Andisols, formed by weathering of volcanic ash under well-drained conditions, dominated location 6; they are characterized by low bulk density (Table 3) and are favorable for keeping aerobic conditions [25].

Larger *ρ*_b_ was observed at 80 cm depth than at 20 cm depth across the six locations except at location 4 (Table 3). The opposite was expected for *θ*_t_, given the inverse relationship between *θ*_t_ and *ρ*_b_. Smaller *ρ*_b_ had resulted in larger *θ*_t_ given their inverse relationship (Equation (1)). At the 20 cm depth, *ρ*_b_ ranged from 0.49 at locations 6 to 0.95 g cm^−3^ at location 4; however, at 80 cm depth, it ranged from 0.72 at location 6 to 1.27 g cm^−3^ at location 1. At the 20 cm depth, *θ*_t_ ranged from 0.66 at location 4 to 0.72 cm^3^ cm^−3^ at location 5; whereas, at 80 cm depth, it varied between 0.56 at location 1 and 0.67 cm^3^ cm^−3^ at locations 4.

The values of EC at 20 cm soil depth were almost double of those at 80 cm soil depth at locations 1 through 3; whereas, at locations 4 and 5, the EC values at 20 cm soil depth were 1.5 and 1.2 times those at 80 cm soil depth, respectively (Table 3). At 20 cm soil depth, the EC ranged from 426 at location 3 to 2,016 μS cm^−1^ at location 1; whereas, at 80 cm soil depth, it ranged from 222 at location 3 to 1,270 μS cm^−1^ at location 4. Decomposition of the organic matter from the tree litter might have resulted in the larger EC values of the soil samples of the 20 cm soil layer. Reduction in pH of the top soil layers at locations 1 through 3 might have also resulted from the decomposition of organic matter. The soils from the two depths of the six locations were acidic to neutral as their pH varied from 4.24 at 20 cm depth at location 2 to 5.81 at 20 cm depth at location 5.

ANOVA results showed a significant (*P* < 0.05) increase in *ρ*_b_ and EC values and a significant (*P* < 0.05) decrease in *θ*_t_ with increase in soil depth (Table 4). This may be due to compaction as a result of the overburden from the above soil load. Soil compaction enhanced *ρ*_b_ and thus reduced *θ*_t_ as shown by Equation (1). There was no significant effect of soil depth on CC and soil pH. Location had a significant (*P* < 0.05) effect on *ρ*_b_, *θ*_t_, and EC and a highly significant (*P* < 0.01) effect on CC.

### 3.2. Spatial Variability of Bulk Density and Total Porosity

**Figure 2.**
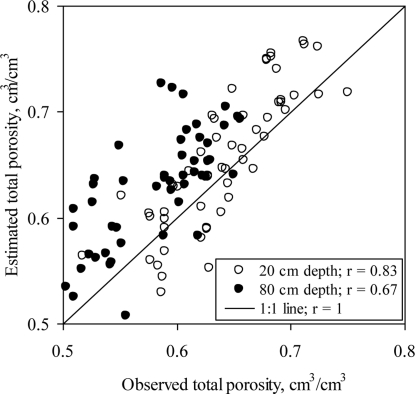
Correlation between the actual total porosity at the two depths (20 and 80 cm) and that estimated from the relationship *θ*_t_ = 1 − *ρ*_b_/*ρ*_s_, where *θ*_t_ is total porosity, *ρ*_b_ is bulk density, and *ρ*_s_ is the particle density (2.65 g cm^−3^).

In general, major soil properties including *ρ*_b_ and *θ*_t_ are consistent for a given soil type, series or order. Variations in *ρ*_b_ and *θ*_t_ are due to many factors such as high organic matter, CC or both. Most of the shrink-swell clays exhibited variability in *ρ*_b_ and *θ*_t_. The shrink-swell behavior of the soils at locations 1 through 5 was confirmed from the good agreement between the actual *θ*_t_ and those calculated from Equation 1 (Figure 2). Small CC at location 6 suppressed soil shrinking and swelling at this location resulting in a weak correlation (*r* < 0.4) between the actual and calculated *θ*_t_.

Pearson’s correlation test was conducted to correlate *ρ*_b_ and *θ*_t_ at 20 and 80 cm depths. Overall, there was a non-significant inverse relationship between *ρ*_b_ and *θ*_t_ except at 20 cm depth where a stronger (*r* = −0.93) inverse significant (*P* < 0.05) relationship existed. The strongest (*r* = 0.95) significant (*P* < 0.05) relationship between *ρ*_b_ at 20 and 80 cm depths reflected the increasing trend of *ρ*_b_ with soil depth at all experimental locations.

### 3.3. Soil Water Content Dynamics due to Spatial Variability of Bulk Density and Total Porosity

**Figure 3.**
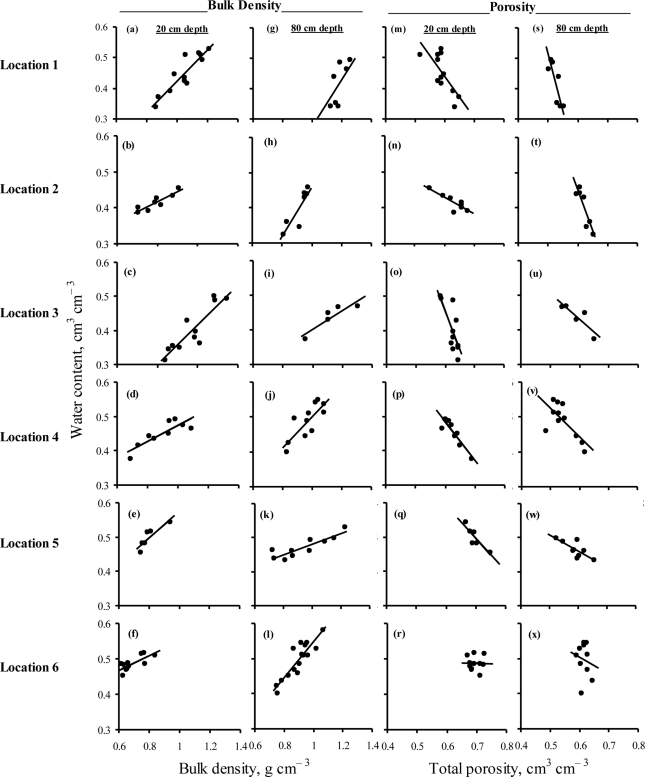
Effect of spatial variability of soil bulk density (figures on left) and actual total porosity (figures on right) on field measured soil water content.

There was a linear increase in *ρ*_b_ and a linear decrease in *θ*_t_ with increase in soil water content at all depths and locations (Figure 3). Slopes of the *ρ*_b_—water content and *θ*_t_—water content models for the two depths and six locations were non-uniform reflecting a great spatial variability in these properties. This was an indicator of great soil water content dynamics due to shrinking and swelling—induced changes in *ρ*_b_ and *θ*_t_. Such behavior of water content dynamics emphasized the need for site-specific calibration equations for soil water content sensors. There were weak *θ*_t_—water content relationships (*r* = 7E-04, 0.02) at location 6 [Figures 3(r,x)] partially due to high ash and organic matter contents and partially due to poor structure of highly weathered soil (personal observations). Volcanic ash soils are classified as an Andisol with low densities and assumption of *ρ*_s_ = 2.65 g cm^−3^ may not be valid for these soils [25]. Moreover, Andisol usually have peculiar permittivity relations due to high surface area and low densities [26–30]. Our results showed that variations in *ρ*_b_ can affect soil water content estimation of high CC soils due to their shrink-swell behavior. Fares *et al.* [31] and Polyakov *et al.* [32] reported similar results for a sandy clay loam soil and for a weathered clay loam soil, respectively. Yule [33] and Smith [34] have reported errors in volumetric water content calculation made from *ρ*_b_ information when the *ρ*_b_ was not determined at the right moisture content.

### 3.4. Field Calibration of Sensors

Since soil depths and locations had significant effect on *ρ*_b_, *θ*_t_, CC, and EC (Table 4) in addition to the significant effect of *ρ*_b_ and *θ*_t_ on soil water content dynamics (Figure 3), field calibration equations for each sensor were established for each depth and location (Table 5). These site-specific field calibration equations of the EC-20, ML2x, and SM200 accurately predicted the actual water content (*r* > 0.88; RMSE 0.011 to 0.054 cm^3^ cm^−3^), compared with their respective default equations (*r* > 0.86; RMSE 0.026 to 0.247 cm^3^ cm^−3^). The values of RMSE and MBE of the actual water content and the calculated water content using default equations are inside the parentheses in Table 5. The values of MBE for the site-specific field calibration equations were smaller than those of their corresponding default equations indicating that the site-specific field calibration equations had significantly improved the accuracy of soil water content measurements of these sensors. On the other hand, the default calibration equations substantially under-estimated (larger absolute negative value of MBE) the actual water content compared with their corresponding site-specific field calibration equations (smaller MBE).

The watershed-scale field calibration equations (one equation of each sensor for all depths and locations) of the three sensors seem to substantially (*P* < 0.001) improve the accuracy of the tested sensors (Table 6). Estimation of actual water content using these new equations resulted in smaller RMSE and MBE as compared with their corresponding default equations. The ML2x exhibited the highest improvement in its accuracy with the use of the new calibration equation (*r* = 0.81; RMSE 0.038 cm^3^ cm^−3^) over its default equation (*r* = 0.79; RMSE 0.189 cm^3^ cm^−3^).

Similar improvement in the accuracy of the SM200 was achieved with the watershed-scale field calibration equations (RMSE 0.042 cm^3^ cm^−3^) compared with the default equation (RMSE 0.155 cm^3^ cm^−3^). There was a better agreement between actual water content and that measured by the SM200 watershed-scale field calibration equation (MBE −0.001 cm^3^ cm^−3^) than between actual water content and that determined by the default equation (MBE −0.126 cm^3^ cm^−3^). EC-20 showed the least improvement in its accuracy among the tested sensors with the use of watershed-scale field calibration equation (RMSE 0.054 cm^3^ cm^−3^; MBE −0.034 cm^3^ cm^−3^) compared to that of its default equation (RMSE 0.068 cm^3^ cm^−3^; MBE −0.014 cm^3^ cm^−3^).

**Figure 4.**
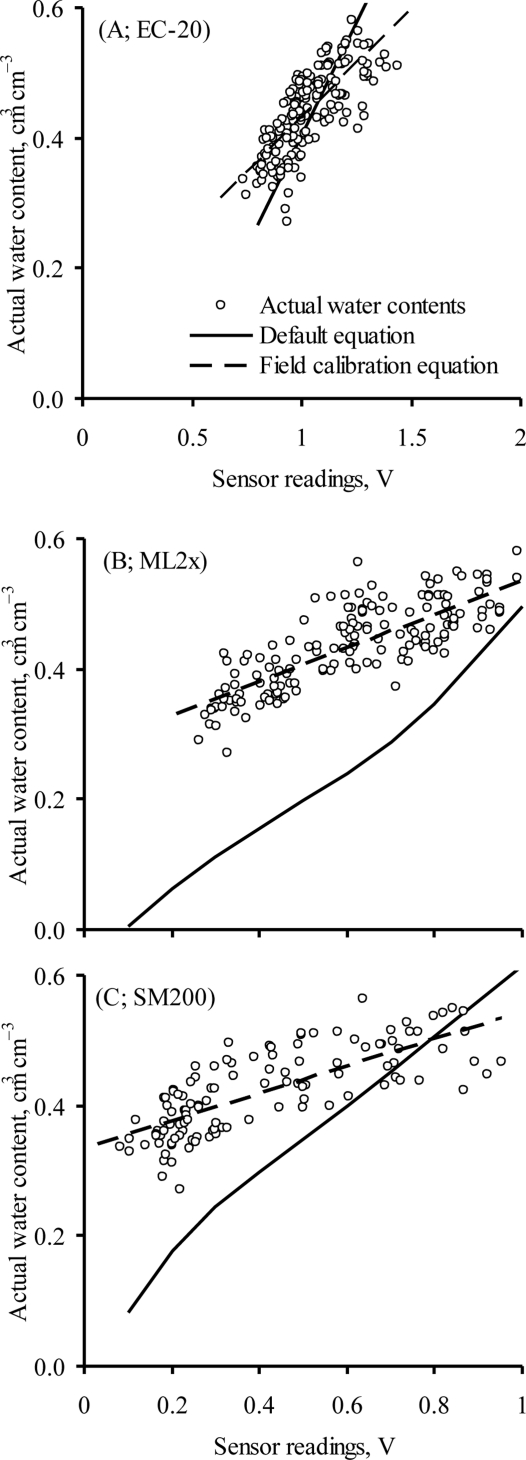
Field measured actual volumetric water content versus sensors’ readings plotted with default and site-specific field calibration models for (**A**) EC-20; (**B**) ML2x; and (**C**) SM200.

The performance of the default and watershed site-specific calibration equations in tracking the actual water content is detailed in Figure 4. There was a minimal difference between the performance of the field and the default (MBE −0.034 cm^3^ cm^−3^ *vs.* −0.014 cm^3^ cm^−3^) equations of EC-20 (Figure 4(a); Table 6).

## 4. Conclusions

The effect of spatial variability of *ρ*_b_, *θ*_t_, CC, and EC on the performance of EC-20, ML2x, and SM200 sensors installed at 20 and 80 cm depths on six locations across the forested upper Mākaha Valley watershed in O’ahu (Hawai’I, USA) was studied. The studied soil properties significantly varied as a function of the water content monitoring depths and locations. Field calibration equations for the three tested sensors improved their performance for accurate measurement of actual soil water content.

The default equations of the ML2x and SM200 showed substantial under-estimations of actual water content; whereas, the field calibration equations of these sensors substantially improved their accuracy of measuring actual water content. The EC-20 default equation performed better than the default equations of the other two sensors. Moreover, there was no significant difference in the performance of the default and the field calibration equations of EC-20. EC-20 had the least improvement in its accuracy with the use of field calibration equation as compared with the other two sensors. Overall, the field calibration equations were more accurate in estimating soil water content (higher P, *r* and lower RMSE, MBE) than their corresponding default equations. The tested sensors need site-specific calibrations for accurate measurement of actual water content of these highly weathered tropical soils. The field calibration equations can mitigate the effects of varying soil properties and improve the accuracy of the tested sensors.

## Figures and Tables

**Table 1.** Sensors used in this study, their operating frequency, and default calibration equations.

**Sensors**	**Operating frequency, MHz**	**Default Calibration Equation**
EC-20	5	*y* = 0.695*x* − 0.29
ML2x	100	*y* = 0.560*x*^3^ − 0.762*x*^2^ + 0.762*x* − 0.063
SM200	100	*y* = 1.611*x*^5^ − 5.48*x*^4^ + 7.248*x*^3^ − 4.61*x*^2^ + 1.917*x* − 0.071

*y*: actual volumetric soil water content (cm^3^ cm^−3^); *x*: sensor readings (V).

**Table 2.** The elevation, saturated hydraulic conductivity (*K*_sat_), the NRCS soil series, particle size distribution, and the USDA soil classification of the soil samples collected from 20 and 80 cm depths at the six monitoring locations in the upper Mākaha Valley sub-watershed.

**Location**	**Elevation, m**	***K*_sat_, cm h^−1^**	**Depth, cm**	**NRCS Soil Series**	**Clay**	**Sand**	**USDA Soil texture**
					**g kg^−1^**		
1	343	90.2	20	Mollisol	313	269	Clay loam
			80	Mollisol	313	269	Clay loam
2	477	10.8	20	Inceptisol	610	308	Clay
			80	Inceptisol	690	250	Clay
3	538	31.4	20	Ultisol	521	167	Clay
			80	Ultisol	640	250	Clay
4	601	63.8	20	Ultisol	313	218	Clay loam
			80	Ultisol	288	320	Clay loam
5	609	16.5	20	Ultisol	263	421	Loam
			80	Ultisol	288	661	Sandy loam
6	725	19.3	20	Andisol	157	371	Loam
			80	Andisol	230	218	Silt loam

**Table 3.** Measured soil bulk density (*ρ*_b_), total porosity (*θ*_t_), electrical conductivity (EC) and pH of the soil samples collected from 20 and 80 cm depths at the six monitoring locations in the upper Mākaha Valley sub-watershed.

**Location**	**Depth (cm)**	***ρ*_b_ g (cm^−3^)**	***θ*_t_ cm^3^ (cm^−3^)**	**EC^#^ μS (cm^−1^)**	**pH^#^**
1	20	0.93	0.69	2016	5.01
	80	1.27	0.56	840	5.52
2	20	0.86	0.67	802	4.24
	80	1.06	0.60	480	4.29
3	20	0.83	0.67	426	4.60
	80	0.97	0.58	222	4.69
4	20	0.95	0.66	1888	5.65
	80	0.93	0.67	1270	4.91
5	20	0.69	0.72	1220	5.81
	80	0.93	0.61	1024	4.89
6	20	0.49	0.80	1189	4.88
	80	0.72	0.75	959	5.09

# Electrical conductivity and pH measurements were made on 1:2 soil:water solutions.

**Table 4.** Results of the factorial general analysis of variance for bulk density, porosity, and clay contents, electrical conductivity, and pH as a function of soil depths and locations.

**Factors**	**Bulk Density**	**Total Porosity**	**Clay Contents**	**Electrical Conductivity**	**pH**
Depth	0.0125*	0.0153*	NS	0.0336*	NS
Location	0.0220*	0.0463*	0.0026**	0.0355*	NS
Interaction	NS	NS	NS	NS	NS

*: significant;

**: highly significant; NS: not significant.

**Table 5.** Equation parameters and statistical indices for the mean square error (RMSE) and mean bias error (MBE) from the comparison of actual water content sensor equations in parentheses.

**Location**	**Depth**	**Sensor**	***a*^¶^*×* 10^−2^**	***b*^§^*×* 10^−2^**	***r***	**RMSE ÷ 10^2^, cm^3^ cm^−3^**	**MBE ÷ 10^2^, cm^3^ cm^−3^**	***n***
1	20	EC-20	0.04	−1.71	0.954(0.945)	3.63 (12.0)	2.82 (10.0)	16
		ML2x	48.68	20.31	0.917(0.919)	3.00 (24.7)	−0.15 (−24.5)	16
		SM200	30.10	29.85	0.968(0.974)	3.73 (14.9)	0.00 (−13.3)	16
	80	EC-20	0.04	−0.20	0.916(0.917)	3.87 (7.31)	2.81 (4.45)	12
		ML2x	47.08	17.59	0.913(0.914)	2.67 (21.3)	0.00 (−21.1)	12
		SM200	24.97	28.25	0.911(0.903)	2.70 (11.5)	−0.00 (−8.63)	12

2	20	EC-20	0.03	11.51	0.917(0.917)	1.79 (7.19)	−0.33 (−4.65)	16
		ML2x	31.74	24.28	0.944(0.937)	1.45 (20.7)	−0.03 (−20.5)	16
		SM200	27.05	32.38	0.930(0.939)	1.62 (19.9)	−0.00 (−19.0)	16
	80	EC-20	0.05	−7.17	0.929(0.928)	3.60 (2.61)	3.19 (−0.39)	12
		ML2x	26.0	25.01	0.929(0.926)	1.63 (18.1)	−0.00 (−17.7)	12
		SM200	18.39	31.25	0.960(0.957)	1.59 (13.4)	1.00 (−10.3)	12

3	20	EC-20	0.04	−2.30	0.933(0.933)	5.40 (6.04)	−4.75 (−2.45)	16
		ML2x	55.24	13.01	0.921(0.919)	2.79 (21.3)	−0.08 (−21.0)	16
		SM200	27.05	32.38	0.921(0.922)	2.80 (12.7)	−0.00 (−12.3)	16
	80	EC-20	0.04	0.12	0.935(0.935)	1.66 (5.10)	1.26 (4.46)	10
		ML2x	19.49	28.66	0.920(0.925)	1.18 (13.8)	−0.00 (−13.0)	10
		SM200	42.16	24.84	0.891(0.891)	1.36 (9.28)	0.00 (−0.14)	10

4	20	EC-20	0.04	15.1	0.909(0.909)	2.14 (6.13)	1.18 (−2.16)	18
		ML2x	24.25	32.0	0.884(0.877)	1.99 (23.8)	0.27 (−23.4)	18
		SM200	17.64	38.07	0.878(0.890)	2.04 (18.4)	−0.00 (−16.5)	18
	80	EC-20	0.04	−0.12	0.917(0.917)	1.61 (5.78)	−16.0 (−4.08)	14
		ML2x	38.53	21.79	0.883(0.864)	0.75 (19.6)	−0.00 (−19.4)	14
		SM200	16.81	40.36	0.917(0.912)	3.50 (16.5)	−0.95 (−13.8)	14

5	20	EC-20	0.03	15.02	0.908(0.908)	2.21 (6.83)	−0.53 (−2.62)	12
		ML2x	60.96	−1.05	0.897(0.888)	3.08 (13.1)	2.10 (−12.9)	12
		SM200	–	–	–	–	–	–
	80	EC-20	0.04	−4.12	0.911(0.911)	10.7 (6.18)	−10.6 (−5.38)	16
		ML2x	33.44	18.39	0.903(0.910)	1.82 (8.91)	0.17 (−7.73)	16
		SM200	–	–	–	–	–	–

6	20	EC-20	0.04	−0.20	0.942(0.942)	12.3 (6.83)	−12.2 (−5.73)	18
		ML2x	31.09	20.04	0.906(0.886)	0.95 (9.02)	0.00 (−8.68)	12
		SM200	–	–	–	–	–	–
	80	EC-20	0.06	−9.29	0.961(0.961)	5.26 (4.93)	5.10 (−4.66)	16
		ML2x	26.51	30.51	0.943(0.953)	1.08 (16.5)	−0.00 (−15.9)	7
		SM200	–	–	–	–	–	–

¶ slope;

§ *y* intercept.

**Table 6.** Regression parameters of watershed-specific field calibration equations and accuracy indicators (RMSE; MBE; mean bias error) of field and default equations.

**Sensor**	***N***	**Calibration**	***a*^¶^**	***b*^§^**	**P**	***r***	**RMSE ÷ 10^2^, cm^3^ cm^−3^**	**MBE, ÷ 10^2^, cm^3^ cm^−3^**
EC-20	176	Field	0.3	0.0969	4.24E-33	0.75	5.41	−3.36
		Default	0.695	−0.29	4.24E-33	0.75	6.84	−1.37

ML2x	160	Field	0.2592	0.2768	1.41E-38	0.81	3.79	0.002
		Default	#	#	1.34E-35	0.79	18.9	−17.7

SM200	113	Field	0.2109	0.3334	2.06E-22	0.75	4.22	−0.103
		Default	#	#	4.46E-23	0.76	15.5	−12.6

¶ slope;

§ *y* intercept;

#: Parameters are given in Table 1.
